# Benchmarking Dataset of Signals from a Commercial MEMS Magnetic–Angular Rate–Gravity (MARG) Sensor Manipulated in Regions with and without Geomagnetic Distortion

**DOI:** 10.3390/s23083786

**Published:** 2023-04-07

**Authors:** Pontakorn Sonchan, Neeranut Ratchatanantakit, Nonnarit O-larnnithipong, Malek Adjouadi, Armando Barreto

**Affiliations:** Electrical and Computer Engineering Department, Florida International University, Miami, FL 33174, USA; psonc001@fiu.edu (P.S.); nratc001@fiu.edu (N.R.); nolarnni@fiu.edu (N.O.-l.); adjouadi@fiu.edu (M.A.)

**Keywords:** MARG, MIMU, orientation estimation, sensor fusion algorithm, dataset, orientation algorithm benchmarking

## Abstract

In this paper, we present the FIU MARG Dataset (FIUMARGDB) of signals from the tri-axial accelerometer, gyroscope, and magnetometer contained in a low-cost miniature magnetic–angular rate–gravity (MARG) sensor module (also known as magnetic inertial measurement unit, MIMU) for the evaluation of MARG orientation estimation algorithms. The dataset contains 30 files resulting from different volunteer subjects executing manipulations of the MARG in areas with and without magnetic distortion. Each file also contains reference (“ground truth”) MARG orientations (as quaternions) determined by an optical motion capture system during the recording of the MARG signals. The creation of FIUMARGDB responds to the increasing need for the objective comparison of the performance of MARG orientation estimation algorithms, using the same inputs (accelerometer, gyroscope, and magnetometer signals) recorded under varied circumstances, as MARG modules hold great promise for human motion tracking applications. This dataset specifically addresses the need to study and manage the degradation of orientation estimates that occur when MARGs operate in regions with known magnetic field distortions. To our knowledge, no other dataset with these characteristics is currently available. FIUMARGDB can be accessed through the URL indicated in the conclusions section. It is our hope that the availability of this dataset will lead to the development of orientation estimation algorithms that are more resilient to magnetic distortions, for the benefit of fields as diverse as human–computer interaction, kinesiology, motor rehabilitation, etc.

## 1. Introduction

### 1.1. Need for a MEMS MARG Benchmarking Dataset

One of the earliest developments of a micro-machined accelerometer was reported by Roylance et al. in 1979 [1]. However, it would be about 15 years before these devices were embedded into end-user products and manufactured in large volumes [2]. By 2009, miniature gyroscopes, also developed as micro electro mechanical systems (MEMs) would become commercially available as well [3]. Both of these sensors were then packaged commercially as “6-degrees-of freedom” MEMS inertial measurement units (IMUs), sparking significant interest due to the advantages that these sensor modules could have in many prospective applications. These modules were small in size and low in weight, power consumption and heat dissipation, while simultaneously offering measurements of acceleration along three orthogonal axes and rotational speed about those same axes. In principle, these were the same measurements required in aeronautical and maritime inertial navigation systems that use the “strapdown configuration”. The strapdown approach estimates orientation by integration of the gyroscope measurements and utilizes it for the appropriate projection (“resolution”) of accelerometer readings to an inertial coordinate frame with one of its axes parallel to the gravitational acceleration [4,5].

For the strapdown approach, the orientation estimate must be very accurate, so that the acceleration of gravity can be correctly discounted from the resolved accelerometer readings and then double-integrated to yield a position estimate. Unfortunately, the use of MEMS IMUs has shown that the quality of the acceleration and rotational speed measurements from these miniature sensors is significantly poorer than that of their navigational or strategic counterparts [6,7,8]. In response, researchers have focused efforts on the development of orientation estimation approaches that can be resilient to the imperfections of signals from the MEMS accelerometers and gyroscopes. In particular, the offset frequently found in the outputs from MEMS gyroscopes is highly damaging to any process that uses those signals for orientation estimation. This is because rotational speeds must be iteratively accumulated (integrated) in one form or another to keep a running tally of total rotation from a starting orientation, i.e., the quantification of the current orientation of a rigid body with respect to its initial orientation. The offset in the outputs of MEMS gyroscopes varies from “run-to-run” and it even experiences significant “in-run” fluctuations [7]. This makes complete and permanent cancellation of gyroscope offsets extremely difficult to achieve and gives rise to orientation “drift” errors when not properly counteracted.

Accordingly, many new MEMS orientation estimation approaches strive to implement some form of information fusion, so that the accelerometer readings may be used to enhance (often correct) initial orientation estimates obtained from gyroscopic readings. In this spirit, the “6-degrees-of-freedom” IMUs were augmented with tri-axial magnetometers incorporated first on the same module enclosure and later as additional components in the same chip as the accelerometer and gyroscope. These are the sensor modules that we prefer to designate as “magnetic–angular rate–gravity” (MARG) modules, although they are sometimes identified also as “9-degrees-of-freedom” IMUs or magnetic and inertial measurement units (MIMUs). For the last several decades, researchers have proposed multiple approaches to solve the problem of real-time MARG orientation estimatation, attempting to fulfill the promise that MEMS IMUs first seemed to have in the earlier XXI century. In their 2021 paper, Nazarahari and Rouhani summarized the breadth and depth of the different approaches proposed over the last 40 years [9], finding that there may not be a single existing algorithm that outperforms the rest under all conditions. Instead, they found that certain algorithms seem to perform better when specific considerations (e.g., latency, computational complexity, characteristics of the environment in which the MARG is used, etc.) are given priority. Accordingly, when they identify the future research challenges in the field, they state “… we suggest that test platforms and benchmarking studies are required to identify the most effective SFAs (Sensor Fusion Algorithms), as well as techniques that could improve the accuracy and robustness of SFAs.” [9].

Useful application of MEMS MARG modules in areas such as human–computer interactions and human motion studies requires significant accuracy and robustness in the MARG orientation estimation algorithms used. For example, if a physical hand-held controller with a single MEMS MARG attached to it is used as a ray-casting pointer for three-dimensional virtual environments (e.g., [10]), orientation estimation errors between the actual orientation of the hand-held controller and the orientation of the virtual ray created will be detrimental to the task when pointing to objects at increasing virtual depths, as a virtual object will span a smaller amount of degrees of visual field when it is simulated at increasing virtual depth (away from the user). Conversely, any orientation error will be projected to a longer error distance when the ray intersects deeper planes in the virtual environment. In more complex uses of MARG modules for human–computer interactions, such as their utilization in instrumented gloves for real-time hand tracking and gesture identification (e.g., [11]), each finger is frequently modeled as a kinematic chain. Therefore, the position of the fingertip is computed as a composition of the lengths and orientations of all three finger segments (proximal phalanx, intermediate phalanx, and distal phalanx), as estimated by the MARGs attached to them. If each MARG orientation estimate contains errors, the estimated position of the fingertip may be significantly flawed, resulting in an unacceptable internal representation of the user’s hand in the computer. Furthermore, developing techniques that will allow a user to “grab” virtual objects with their virtual hand will demand a high level of accuracy in the virtual representations of position and orientation of all hand segments.

In principle, MARG module orientation estimation is possible based on gyroscope data, particularly when periodically corrected by information from the accelerometer and the magnetometer. However, a critical challenge that emerges is the risk of applying a correction when the assumptions made for the utilization of secondary sensor (accelerometer or magnetometer) data are not met [12]. If such inappropriate corrections are fully applied, significant error may be introduced in the current and future orientation estimates.

For magnetometer corrections, the assumption is, typically, that the geomagnetic vector has the same magnitude and the same direction throughout the complete operational space in which the MARG will be used. Indeed, the geomagnetic vector, considered in isolation, would be constant in magnitude and direction within the reduced environments (e.g., a room or even a city) in which these types of MARG applications will take place. However, in the modern built environment, it is essentially unavoidable to find ourselves near large ferromagnetic objects, such as metal furniture and even structural elements embedded in our dwellings, laboratories, and buildings. These objects could have high magnetic permeability that may cause the “bending” of the magnetic field lines, yielding localized distortions of the magnetic field in their neighborhood. As a result, possible local geomagnetic distortions cannot be overlooked in the evaluation of MARG orientation estimation algorithms.

Accordingly, we believe that there is a need for benchmarking datasets that challenge the MEMS MARG orientation algorithms in different ways. Specifically, the study of the resilience of MARG orientation algorithms that use magnetometer readings of local distortions of the geomagnetic field has attracted a lot of attention in recent years and should be fostered by the creation of calibrated (i.e., containing ground truth orientations) datasets collected in spatial regions where the geomagnetic field is both normal *and* distorted. The impact of magnetic field disruptions on MARG orientation algorithms within fields such as biomechanics research and human–computer interaction became particularly clear after the publication of the 2009 paper by deVries et al. [13], who advised researchers to “‘Map’ your laboratory on ferromagnetic characteristics…” and “Preferably use IMUs well away from floors, walls, and ceilings”. These limiting concerns were echoed more recently (2019) by Picerno in his comprehensive survey of techniques for studying joint kinematics by using inertial and magnetic sensors [14]: “Unfortunately, the presence of ferromagnetic disturbances distorts the sensing of the local magnetic north. This negatively affects the reliability of the estimated sensor’s orientation and may, thus, compromise the usability of such application in the clinical settings, which are normally characterized by ferromagnetic materials and related interferences.”

The perceived need for a calibrated dataset that may be used for benchmarking the performance of MARG orientation algorithms in regions with and without magnetic distortions has prompted us to develop the FIU MARG Dataset (FIUMARGDB), which we introduce in this paper.

### 1.2. Related Datasets and Studies

As it became clear that different MARG orientation estimators exhibited performance advantages for different scenarios, and that their “tuning”, i.e., the assignment of specific numerical values to operational parameters of the estimator, was critical, a more urgent need for testing common data emerged. One of the earliest data repositories created specifically for MARG evaluation was “RepoIMU”, developed by Szczesna et al. from The Silesian University of Technology, Gliwice, Poland, in 2016 [15]. The paper that presented the dataset expresses the authors’ belief that, at the time, “A similar repository was not found.” While the authors note three contemporary datasets (one for the study of pedestrian navigation [16], one for human activity recognition [17], and one that combines readings from five Xsens inertial measurement units and data from a Kinect system [18]), they make a compelling case to conclude that none of those datasets would be truly appropriate for benchmarking the performance of MARG sensor modules. The third dataset references is, perhaps, the closest to the kind of dataset needed for MARG orientation algorithm benchmarking, but includes data from the Kinect system as “ground truth”, which is known to compare poorly in accuracy and stability to data available from a multicamera motion capture system.

The RepoIMU repository includes recordings from IMU sensor(s) and a Vicon Nexus optical motion capture system under two scenarios: (a) movement of a “wand” (T-stick) with one Xsens MTi-G-28 A53 G35 MARG (in this case, the Vicon system used six markers) and (b) movement of three custom-built IMUs mounted on each of the three sections of an articulated pendulum (in this case, the Vicon system used eight markers). Each wand experiment was reported in a single file. Each pendulum experiment resulted in three separate files (one for each of the IMU modules). The files are comma-separated value (CSV) files with headers in the first row. Each file includes records (rows) comprising a timestamp (in milliseconds) and tri-axial accelerometer, gyroscope, and magnetometer data from the corresponding IMU, as well as quaternion orientation estimates for the corresponding segment. The repository contains a total of 95 recordings, and is available in GitHub [19].

The paper implies that the IMU readings and the Vicon system readings were initially recorded to separate files, as a detailed description of the “data synchronization” process that needed to be performed is included. In fact, the paper mentions that the Vicon system was operated always at a sampling frequency of 100 Hz, whereas the sampling frequency for the IMU signals was different for different tests (as shown in Table 2 of the RepoIMU paper, which shows values of 90 or 166 Hz). In addition, the IMUs used are not necessarily the same type as the low-cost miniature MARG boards that are available at the time of writing (e.g., 3-Space™ Nano IC, from Yost Labs, 3.8 mm × 5.2 mm × 1.1 mm, under USD 20 in large quantities [20]). The Xsens MTi g modules are more complex, combining a MEMS IMU, a GPS, and a barometer, with dimensions of 58 × 58 × 33 mm [21]. The characteristics of the custom IMUs used in the pendulum recordings are not necessarily similar to the characteristics of contemporary low-cost miniature MARG chips. Furthermore, the RepoIMU dataset did not control or insert any form of known magnetic field interference as part of the test scenarios.

In the last 2 years, the need for an objective and detailed comparison of the performance of the many available MEMS MARG estimators has prompted researchers to apply some of the leading estimators to the same sets of MARG data, which they have collected for that purpose. In some cases, researchers have made the data available to other interested parties. That is the case in the study of the accuracy of ten orientation estimation algorithms performed by Caruso et al. (Politecnico di Torino, Bio Robotics Institute in Pisa, University of Berlin and University of Sassari) [22]. The aim of their work was to analyze the accuracy of ten orientation estimators (called sensor fusion algorithms, SFAs), across a matrix of three rotation rates (slow, medium, and fast) by three (pairs of) commercial MARG modules: Xsens-MTx, APDM-Opal, and Shimmer-Shimmer3. For all recordings, the six MARGs were attached to a wooden board, which also had eight reflective spherical markers on it, so that the reference orientation of the whole board (as a rigid body) could be determined by a Vicon T20—Nexus 2.7 optical motion tracking system involving 12 cameras. In this case, also the MARG data and the optical motion capture data seem to have been initially recorded in separate files (since the optical motion capture data “were first processed in Nexus 2.7” and the paper describes a two-step process of synchronization). Both the Matlab algorithm implementations and the three Matlab data (*. mat) files with MARG and Vicon data were made available through IEEE Dataport [23].

The main emphasis of this research effort was the implementation, “tuning” (i.e., search for best parameters for each algorithm), and comparison of the algorithms. The creation of the dataset was a means to that end. All three of the MARG modules used were more complex than the low-cost miniature MARG boards most readily available (such as the Yost Labs 3-Space™ Nano IC). The three recording scenarios did not control or insert any form of known magnetic field interference as part of the test environment.

Another MARG dataset, which was also developed in order to perform a comparative assessment of orientation estimation algorithms, is the one used for a broad study comparing the performance 36 orientation estimators (SFAs) performed by Nazarahari and Rouhani from the University of Alberta, Canada [24]. They applied the algorithms to data from three Xsens MTws MIMUs attached to rigid plastic plates equipped with four retro-reflective markers and fixed to the subject’s foot, shank, and thigh. The reflective markers were tracked by an eight-camera Vicon motion capture system. The recordings involved the participation of nine able-bodied participants. Each participant performed actions in two phases; phase I (DataShort.mat records) included standing and brief episodes of walking, turning, jumping, and hopping in order to explore various motion patterns and intensities (each complete trial lasted 137 ± 7 s.) and phase II (DataLong.mat records) included standing and longer intervals of walking and turning to explore highly dynamic long-duration tasks (each complete trial lasted 393 ± 3 s.)

The Xsens MTws are closer in size to the miniature MEMS MARG chips (e.g., Yost Lab’s 3-Space™ Nano IC), but they are commercialized in a different price range. In this effort, the main focus was on the implementation, tuning, and comparison of the algorithms, but the associated data (from Subject 2) remain available (as Matlab data files) on the website of the Neuromuscular Control and Biomechanics Laboratory of the University of Alberta [25], and include the ground truth foot, shank, and thigh orientation results (as quaternions) from the Vicon system. In a paper that describes the comparison of the algorithms, no mention is made of the introduction or control of known magnetic distortions, other than the decision to implement the SFA algorithms using the foot-worn MIMU only because it “was close to the ground surface and experienced the highest magnetic disturbance compared to shank/thigh MIMUs, according to De Vries et al.”.

Previous studies and datasets have not specifically established contrasting recording conditions that would expose the MARG to environments with and without magnetic disturbances. There have been studies where those contrasting magnetic conditions were studied, but their authors have not made the corresponding MARG datafiles accessible to other interested parties. Roetenberg et al. [26,27] studied short MARG records where an Xsense MT9 MARG was rotated in alternating locations that they characterized as “free space” and “close to 3.75 kg of metal” [26]. Subsequently, they performed three types of tests [27]. They first studied the magnetic disruption effect on signals from a static Xsens MT9 MARG as “an iron cylinder of 3.75 kg was placed near the sensor module for 10 min without moving the sensor”. A second series of 10 quasi-static tests included rotations of + and −90° performed along the three axes. “After these rotations, the iron cylinder was placed at 5 cm of the module and a new sequence of rotations was performed in opposite directions. The iron was then taken away and the sensor was rotated 90° along the x axis and −90° back.” In the third experiment, three 10 cm carbon fiber sticks with optical markers in their ends were attached at orthogonal directions on the MARG and the assembly was attached to a 50 cm long stick “and moved by hand near a large iron tool case”. This allowed recording of the MARG signals while an orientation reference (ground truth) was obtained by a Vicon 370 3-D optical tracking system with six cameras. “The movements consisted of small and large rotations along multiple axes at different velocities and different distances from the ferromagnetic case.”

In these studies, the data were utilized internally by Roetenberg’s research group to develop and evaluate their magnetic disturbance compensation approach, in which varying weight is assigned to the contributions of the magnetometer signals in the correction stage of a Kalman filter. The emphases of these studies and publications were on the crafting of the enhanced Kalman filter orientation estimator and no mention was made of availability of the MARG data to external parties.

The growing interest in comparing the performance of diverse MEMS MARG orientation algorithms and the lack of available datasets that include recording situations that deliberately recorded MARG signals in regions with and without geomagnetic field disruptions has prompted us to develop the FIUMARGDB dataset. Our intent was to collect recordings partially taken in magnetically disrupted regions that were specifically and purposely set up. We asked volunteers to execute a fixed sequence of pre-specified rotations and translations moving a low-cost, commercially available MEMS MARG module, and we have recorded from multiple volunteers so as to capture the different movement idiosyncrasies that different users of the MARG-based human–computer interaction device could exhibit.

## 2. Materials and Methods

In this section, we briefly describe the setup used for the recording of the files, the MARG module used to record the signals, and the optical motion capture system that was used to simultaneously produce estimates of MARG orientation and position that can be used as “ground truth” for benchmarking the results of multiple MARG orientation algorithms. We also describe in detail the sequence of translations and rotations that the subjects were instructed to execute.

### 2.1. Recording Environment

As the goal was to obtain data recordings where the MARG would be operating in both magnetically undistorted environments and magnetically distorted regions, our initial concern was to set up an area for the recordings that would not (originally) have magnetic distortions. To this end, the three locations in which the MARG would be operated were defined within a region in our laboratory in which we had previously repeatedly mapped the magnetic field at intervals of 1 foot (25.4 cm) in all three orthogonal directions [28]. The three locations where the MARG would operate during the recordings, (H), (A), and (B), were defined in the portion of the previously mapped space where the magnetic field vectors had been found to have the same orientation and magnitude, away from any large ferromagnetic objects. All the necessary supports were made from wood and glued together (avoiding the use of metallic fasteners).

A 3′ (91.44 cm) by 2′ (60.96 cm) poster presentation cardboard was placed horizontally to provide the subjects with a visual plane of reference (although the subjects were instructed to hold the MARG above this reference plane, never allowing the MARG to touch the cardboard, except at the “home location” at the beginning and end of the recording). The reference plane was at an approximate height of 1 m above the floor of the laboratory.

**Figure 1:**
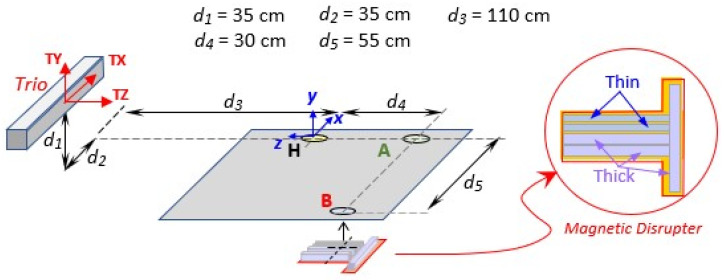
Relative position of locations (H), (A), and (B) with respect to the origin of coordinates for the optical motion capture Trio system. The Trio system uses a right-handed coordinate system (TX, TY, TZ). This is used only for position coordinates. The MARG uses a left-handed coordinate system (x, y, z). (The “Thin” and “Thick” identifiers for the steel bars are explained in Section 2.2.3).

Paper labels with the letters “H”, “A”, and “B” were pasted on the horizontal reference plane to guide the movements executed by the subjects. These three locations were arranged as a capital “L” that had been mirrored along its vertical stroke, with (A) located at the intersection of the two strokes. (H) was located about 30 cm in the approximate direction north (For repeatability, the (H), (A), and (B) locations in our setup were placed on lines that run parallel to the grid defined by the tiles in the floor of our laboratory. That grid is only approximately oriented south–north and east–west.) from (A) and (B) was located about 55 cm in the approximate direction west of (A). The relative distances between locations (H), (A), and (B) are displayed in Figure 1.

Since the series of manipulations instructed to the subjects requires them to start by picking up the MARG from location (H), where it would be resting on a “cradle”, the inertial frame of reference for orientation purposes would naturally be the same as the body frame of reference at that initial moment of the recording (which we will refer here as “startup”). As the MARG we used (see below) adopts a left-handed orthogonal set of axes, that would be also the one naturally used for the (fixed) inertial frame of reference. Those axes are as described in Table 1.

### 2.2. MARG Module, Optical Motion Tracking System, and Magnetic Disrupter Used

#### 2.2.1. MARG Module Used

The MARG sensor used for the recordings was a 3-Space™ Wireless 2.4 GHz Attitude and Heading Reference System (AHRS)/inertial measurement unit (IMU) from Yost Labs, Portsmouth, OH, USA, 45662 (https://yostlabs.com/, accessed on 15 February 2023). We chose to use the 3-Space MARG because the manufacturer makes it available in a wide spectrum of versions, all built surrounding the same basic sensor with different types of enclosures and communication alternatives [29]. This will accommodate widely varying user needs in such a way that no superfluous features need to be purchased. The 3-Space family spans a wide range, from the Nano IC model, a low-cost single surface-mount integrated circuit, to 2.4 GHz wireless or Bluetooth versions and even a watertight USB/RS232 module version. The 3-Space sensor has been validated with calibrated movements performed by an industrial robot and found to be appropriate for a prospective application in a study performing joint angle analyses of surgeons performing laparoscopic surgery [30].

**Figure 2:**
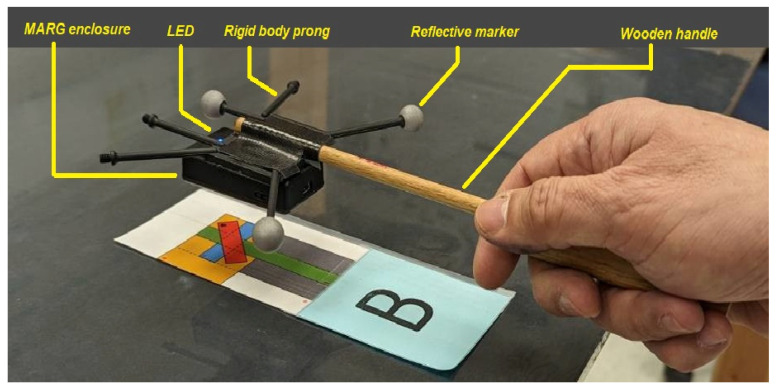
Wand assembly manipulated by the volunteer subjects, shown in Pose 1 <Default Pose> over location (B).

The version of the 3-Space MARG we used was contained in a 60 mm × 35 mm × 15 mm plastic enclosure. The MARG exchanges data and commands with the host personal computer through a matching receiver (“dongle”) connected to a USB port in the host. For our recordings, the MARG enclosure was firmly attached to the center of an OptiTrack (plastic) “hand rigid body”. Three M4 12.7 mm (diameter) reflective spheres were attached to three of the six available prongs of the hand rigid body in such a way that the MARG was located approximately at the center of the triangle defined by the three reflective spherical markers. A lightweight wooden handle was added to the plastic “hand rigid body” so that volunteers could more easily manipulate the MARG. Figure 2 shows the complete wand assembly manipulated by the volunteer subjects.

As mentioned before, the default coordinate axis frame for the MARG we used is an orthogonal (Cartesian) left-handed system, where the positive Z (body) axis points to the LED located at one edge of the module’s largest face (ordinarily lit in blue). Since orientation estimates commonly represent the accumulation of 3D rotations from an initial orientation, the default inertial frame will be considered to match the body frame at startup, and therefore all orientations are indicated as rotations from that initial orientation to the body orientation at any time during the recording. In other words, the orientations of the (body reference frame of the) MARG are referenced to the body frame orientation at startup, which becomes the default (fixed) inertial frame orientation. In all the recording runs, an effort was made to place the MARG in such a way that the initial body frame axes (and therefore the fixed inertial frame axes) matched the directions described in Section 2.1.

The full set of specifications for the 3-Space MARG we used can be consulted in the “3-Space Sensor Miniature Attitude and Heading Reference System With Pedestrian Tracking User’s Manual” (https://yostlabs.com/wp/wp-content/uploads/pdf/3-Space-Sensor-Users-Manual-3.pdf, accessed on 15 February 2023) [29].

It is important to note that, following the native frame of reference of the MARG used, the inertial reference frame used for orientations is as described in Section 2.1. However, the coordinate axis for positions is completely independent of the MARG and will be defined, instead, in accordance with the standards for the optical motion tracking system, described next. This is particularly relevant, as the MARG we used generates internal orientation estimates (as quaternions) utilizing some selectable orientation estimation algorithms. In our files, we also recorded the quaternion orientations generated internally by the MARG using a Kalman filter.

#### 2.2.2. Optical Motion Capture System Used

The optical position and orientation tracking system used during the recordings was the V120:Trio system from OptiTrack (OptiTrack is a company of NaturalPoint, Inc., Corvallis, OR, USA, 97339). This is a system that includes three cameras with infrared filters mounted in a rectangular prism enclosure (58.42 cm × 4.06 cm × 5.08 cm). Each camera is surrounded by a ring with 26 infrared LEDs illuminating the field of view of the cameras. Since the cameras are mounted in the enclosure at the factory, the positional relationships of the camera are pre-calibrated and do not require user intervention. Furthermore, position tracking of an infrared reflective marker (or set of markers defining a rigid body), as well as the orientation of a set of markers defining a rigid body, can be set up simply in the accompanying Motive software (Ver. 2.3.2). A complete list of the technical specifications of the V120:Trio system can be found at (https://optitrack.com/cameras/v120-trio/specs.html, accessed on 15 February 2023) [31].

The longest dimension of the V120:Trio system was placed parallel to the (B)-to-(A) line of our setup, about 140 cm to the north of the (B)-to-(A) line and leveled approximately 35 cm above the reference plane of our setup (poster presentation cardboard).

The OptiTrack Motive software identifies the position of a particular marker within each of the three images captured by the cameras. With precise knowledge of the camera characteristics and the positional relationships between the cameras, the Motive software calculates the three-dimensional px, py, pz position of the spherical marker in a right-handed orthogonal coordinate system which has its origin at the location of the central camara of the Trio device, with its TZ axis coinciding with the optical axis of that camera (pointing towards the scene viewed by the camera). The TX axis for the positions reported by the Trio system runs parallel to the longest dimension of the enclosure. Therefore, with respect to the (H), (A), and (B) labels of our setup, the directions of the Trio axes are specified in Table 2, with the origin of the positional coordinates at the location of the central camera of Trio.

If at least three markers can be detected in the images of the three Trio cameras, they can be designated in Motive as a “Rigid Body”, and the software can then track the position of the geometric center of the triangle and the orientation of a 3D vector from the center of the triangle to one designated marker. For our recordings, the designated marker was selected so that the 3D vector that Trio tracks for orientation runs parallel to the Z axis of the body frame of the MARG. Therefore, at every sampling interval, the Trio system reported the three coordinates, px, py, and pz, of the center of the triangle (according to the axes TX, TY, and TZ) in meters and the orientation of the triangular rigid body as a quaternion. In order to adapt the orientation estimate from Trio to the conventions established for the MARG orientation estimates (for example, the internally generated quaternion that used a Kalman filter for orientation estimations), the following manipulations were applied to define the recorded ***q***_Trio_ quaternion (cam_qx, cam_qy, cam_qz, cam_qw) from the quaternion originally calculated by the Trio system stored in rbData (it must be noted that we used the following ordering of the quaternion components: the 1st, 2nd, and 3rd components are the qx, qy, and qz vector components, respectively. The 4th component is qw, the scalar component).

(1) $$cam\_qx=rbData.qz\times \left(-1\right)$$

(2) cam_qy=rbData.qw

(3) $$cam\_qz=rbData.qx\times \left(-1\right)$$

(4) cam_qw=rbData.qy

The goal of operating the V120:Trio system while the MARG was recording accelerations, rotational speeds, and magnetic field components was to have, for each sampling instant of the MARG sensor data, an independent measurement of the orientation of the MARG (orientation of the rigid body defined by the three spherical reflectors) and its position (the position of the center of the triangle defined by the three spherical reflectors). Accordingly, the orientation calculated by the V120:Trio system (to be referred as the “Trio orientation”, ***q***_Trio_) can act as a “ground truth” for orientation, given its resilience to movement characteristics and magnetic disruptions. Then, the MARG signals can be processed by a variety of MARG orientation algorithms, and their results can be compared to the Trio orientations to assess which algorithm, and under which conditions, yields orientation estimates that more closely resemble the ones from Trio.

The placement of the Trio device was chosen to obtain a good compromise between closeness of the cameras to the markers at any point during the recordings and certainty that the three markers would always remain within the “field of view” of all three cameras. The difficulty of finding an ideal placement for optical motion capture (OMC) systems such as the Trio device is recognized in the motion analysis community. For example, Hindle, Keogh, and Lorimer acknowledge “maintaining a line of sight to each marker throughout the movement is a major challenge when using 3D OMC as markers often become displaced and/or occluded” [32]. We tried a number of combinations of the three distances, d1, d2, and d3, in Figure 1, arriving at the values for these distances shown in the figure. Nonetheless, there can be rare instances during the manipulations performed by the subjects in which the line of sight from any of the three cameras to either one of the spherical markers is obstructed by the MARG holder, or even by another marker. In those cases, the Trio system cannot provide an orientation estimate and repeats the values of quaternion components calculated for the last valid estimation. These events (isTracked = 0) occur in individual sampling instants or in short intervals lasting a few sampling instants and do not distort the overall progression of the quaternion components significantly. Nonetheless, to identify those rare instances, the files also include a flag variable for each sampling instant that is “1” if all the markers were detectable and “0” when at least one of the markers was not detectable. This “isTracked” flag would allow interested users to process the dataset files, applying the interpolation approach of their choice to overwrite the repeated quaternion component values present when “IsTracked” has a value of 0. We have included the Matlab function qTrioFixed = TrioInterp(qTrio, isTracked) in the repository, which performs linear interpolation on the individual quaternion components during the intervals in which “Is Tracked” has a value of 0.

In our data collection setup, both the Trio system and the MARG were connected to the same personal computer host. Our recording software was set to request samples from both systems every 8.3 milliseconds (i.e., at a rate of 120 Hz), and both pieces of information were written simultaneously to a single hard disk file, avoiding the need for after-the-fact synchronization of two different files from each experimental run.

#### 2.2.3. Magnetic Disrupters

One of the priorities in the creation of the files for the dataset was the inclusion, in each experimental run, of both intervals where the MARG would be operating in a magnetically undistorted environment and intervals in which the same device would be subjected to the same type of manipulations but in a region of space known to have distortion of the geomagnetic field. To fulfill the assumption of a uniform, undistorted geomagnetic field in the neighborhood of locations (H) and (A), we defined all three locations for the experiment in a region of space away from furniture that would comprise large ferromagnetic objects. Then, in order to introduce a purposeful magnetic distortion in the neighborhood of location (B), we placed five bars of M35 high speed steel (HSS) from Accusize Industrial Tools, Richmond Hill, Ontario, Canada, under (B), just below the poster presentation cardboard that provided a visual reference plane for the subjects. All five of the steel prisms had a length of 6 inches (15.24 cm), but three of them (“thick”) were 0.5″ × 0.5″ (1.27 cm × 1.27 cm) in cross-section, whereas two of them (“thin”) were 0.25″ × 0.25″ (0.635 cm × 0.635 cm) in cross-section. Both of the “thin” bars and two of the “thick” bars were aligned north-to-south, and the remaining “thick” bar was placed with a west-to-east orientation at the south end of the other four bars, as shown in Figure 1.

### 2.3. Sequence Instructed to the Subjects

With the recording environment set up as described in the previous sections, we asked each of the volunteer subjects to grab the assembly containing the MARG module by the wooden handle and we instructed them to perform a prescribed series of translations and rotations. This series of movements was first demonstrated by one of the experimenters who performed the movements following the slides of a PowerPoint slide show that was being displayed on a computer monitor in front of the subject location, so that the correct sequence of movements would not depend exclusively on the memorization by the subject. Then, the subjects were asked to perform the same sequence of movements with the MARG while they were also shown the PowerPoint slides.

The sequence of movements, listed in Table 1, starts and ends with the MARG resting at the “home location” (H), such that the two largest faces of the MARG enclosure are parallel to the floor and the LED in the enclosure is on the edge that is opposite to the subject, as shown in Figure 2. That initial orientation of the MARG is called the <Default Pose>. After execution of every translation or rotation, the subjects were asked to hold the corresponding “pose” for a count of one to five. It was recommended that, in each one of the poses, the subjects tried to hold the lowest point of the complete MARG holding assembly just below the height of two cigarette packs stacked one top of each other (measured as 44.1 mm). (Two actual cigarette packs were placed on the poster board, away from locations (H), (A), and (B), to serve as a visual reference for the subjects.) Depending on the specific pose, the lowest point of the assembly could correspond to one of the reflective spheres, one of the plastic prongs of the plastic rigid body, etc.

In the <Default Pose>, the axes of the body frame of the MARG are oriented in the same way as the axes of the inertial reference frame, described in Table 1. According to the movement sequence defined in Table 3, only sequence steps 12 to 19, corresponding to Poses 6, 7, 8, 9, and 10, take place in the neighborhood of location (B), i.e., under the influence of a distorted magnetic field (steps 2 and 20 are transitions moving in and then out of the distorted magnetic field). The rotations in each of the steps are specified with respect to the fixed inertial frame axes (following the left-hand frame convention).

The volunteer experimental subjects were recruited from the student body, faculty, and staff of Florida International University. Each subject was given a small enticement (32 GB USB memory or hand-held multimeter) for his/her participation. The experimental procedure was approved by the FIU Internal Review Board (IRB). All subjects were 18 years or older (ages 27.4 ± 7.3 years), without known motion impairments. Each subject held the MARG assembly with their dominant hand. We placed emphasis on including recordings from multiple different human subjects because the database was developed for MARG use in human–computer interaction applications. Therefore, we sought to capture as much as possible of the variability in speeds, trajectories, and stability of poses that can reasonably be found in application of MARG modules within hand-held devices for human–computer communication. This is also the reason why the instructions to the subjects were not exhaustive, leaving room for the idiosyncrasies of movement from each individual. This means that we expected variability in the timing and “accuracy” with which each volunteer subject held the MARG in the instructed poses, which is what would also naturally occur in the ordinary use of a three-dimensional computer interface device (e.g., WiiMote, Nintendo Switch Joy-Con, etc.). This “inaccuracy” on the part of each of the subjects does not impact the intended benchmarking use of the dataset, as the estimation of orientations by new algorithms (even in imperfectly executed poses) will be compared to a “ground truth” estimation of the actual pose held, provided by the Trio system. Our reasons for proposing the orientation estimates from the Trio system as a trustworthy ground truth are detailed in the discussion section (Section 4.1).

A video recording of the sequence of manipulations listed in Table 3, annotated with the identifiers of the poses (P1, …, P10) as they occur, is available as part of the Supplementary Materials for this paper.

### 2.4. Verification of the Magnetic Disruption Established near Location B

We verified that the magnetic field near (B) was disrupted (changed) by the presence of the steel bars measuring the field in the X, Y, and Z directions (according to the blue coordinate axes in Figure 1) both at Location (A) and Location (B). We recorded a file (“LONGRUN.csv”, available in the dataset in folder “Extra_files_1”) in which the MARG started at (H) in the Default Pose (i.e., Pose 1, with the axes of the MARG body frame in the directions indicated by the blue arrows in Figure 1), was translated without rotation to (A), was held there for more than 5 min, was translated without rotation to (B), was held there for more than 5 min, and then it was taken back to (H) without rotation. When we examined the mean and standard deviation in Gauss of the magnetometer readings over 500 consecutive samples, first in (A) and then in (B), we found:

$$\begin{array}{l} MagnetoXYZinA\\ =[\left(-0.0568,0.00013\right),\left(-0.2264,0.00010\right),\left(0.2177,0.00014\right)]\\ MagnetoXYZinB\\ =[\left(-0.0287,0.00110\right),\left(0.0717,0.00120\right),\left(-0.2707,0.00130\right)] \end{array}$$

Here, we can see that all three average magnetometer readings have changed substantially, with the Y and Z components even changing sign, which confirms the magnetic disruption in (B). We also observed that the standard deviations were small (more than one order of magnitude smaller than the averages), which confirms that the readings were essentially constant while the MARG was held in (A) and while the MARG was held in (B). That is, the magnetic disruption at (B) is constant, without variations through time.

## 3. Results

The result of our data collection effort is the compilation of the FIUMARGDB dataset, which contains the simultaneous signals from the MARG sensors and the Trio system for the sequence described in Table 3, executed by 30 volunteer subjects. The length of each record varies, as different subjects executed the sequence of steps in Table 3 at slightly different paces. Amongst the 30 records in the FIUMARGDB dataset, the minimum record lasts 51.50 s and the longest record lasts 153.96 s, with an average of 100.46 s and a standard deviation of 27.21 s. The repository also includes some additional recordings (e.g., the “LONGRUN.csv” record described in the previous paragraph and others), which are found inside folders labeled “Extra_Files_1” to “Extra_Files_4”.

### 3.1. File Organization

Each of the files in FIUMARGDB is a comma-separated value (CSV) ASCII file, where each row contains data collected at a different sampling instant from both the MARG module and the Trio system. The only exception is the very first line in the file, which contains the column headers, also separated by commas.

Table 4 provides the most important aspects of the organization of each file in FIUMARGDB.

The “Confidence Factor” (also described as a “stillness” measure) is a variable computed within the MARG, described as “a value indicating how much the sensor is being moved at the moment. This value will return 1 if the sensor is completely stationary, and will return 0 if it is in motion. This command can also return values in between indicating how much motion the sensor is experiencing.” [29]. Similarly, “isTracked” is a flag that normally takes on the value of 1, indicating successful operation of the Trio system, but may take on the alternative value of 0 if one of more of reflective markers tracked by Trio is not visible.

As shown in Table 4, every file in the FIUMARGDB dataset provides all the elements needed to compare the performance of any given MARG orientation estimation algorithm to the orientation estimates from the Trio system as “ground truth”. Readings from the accelerometer, the gyroscope, and the magnetometer can be fed to the algorithm under study and its output, expressed as a quaternion for each sampling instant, can be compared to the Trio quaternion provided in the file. Entries in columns 9–12 provide the components of the orientation quaternion calculated in real time by the onboard Kalman filter in the MARG, as an example of orientation estimate result.

### 3.2. Visualization of the Contents of a Representative File

**Figure 3:**
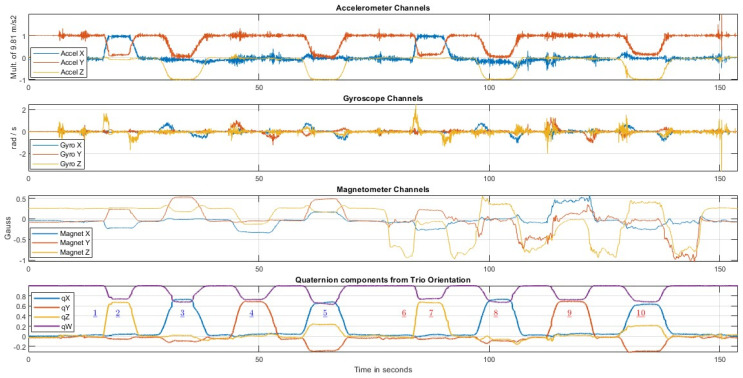
From top to bottom: plot of the three accelerometer channels; plot of the three gyroscope channels; plot of the three magnetometer channels; and temporal evolution of the four components of the rigid body orientation estimate from the Trio system as a quaternion. (Underlined numbers indicate the poses).

**Figure 4:**
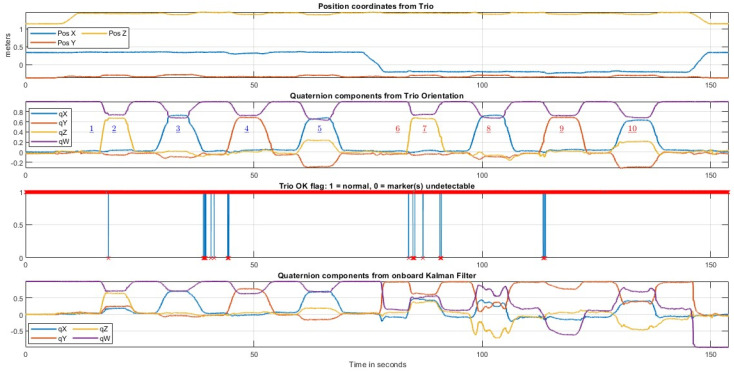
From top to bottom: plot of the three position coordinates with respect to the origin of the Trio frame (TX, TY, TZ); temporal evolution of the four components of the rigid body orientation estimate from the Trio system as a quaternion (converted to the MARGs left-hand frame); plot of the isTracked flag through the recording; and temporal evolution of the four components of onboard Kalman filter quaternion orientation estimate. (Underlined numbers indicate the poses).

**Figure 5:**
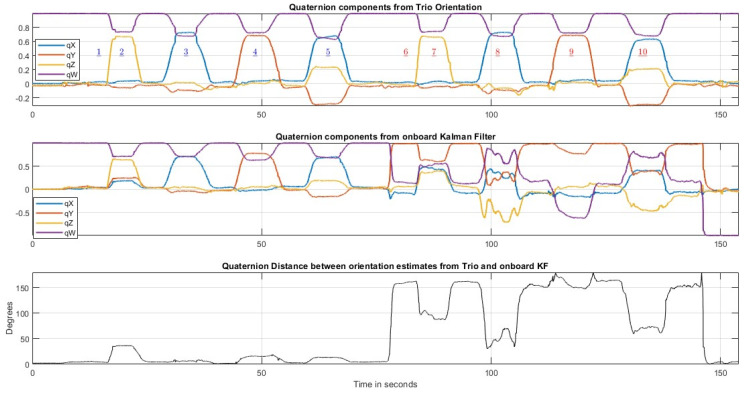
From top to bottom: temporal evolution of the four components of the rigid body quaternion estimate from the Trio system as a quaternion (converted to the MARGs left-hand frame); temporal evolution of the four components of onboard Kalman filter quaternion estimate; and temporal evolution of the “quaternion distance” [33] (angle) between the two orientation quaternions plotted above. (Underlined numbers indicate the poses).

In this subsection, we present the data contained in a representative file (rec03.csv) from the FIUMARGDB dataset. Furthermore, we emphasize in the visualizations how the level of resilience of a given MARG orientation algorithm to magnetic disturbances might be gauged. The FIUMARGDB repository also includes the Matlab functions used to create Figure 3, Figure 4 and Figure 5.

Figure 3 shows the information obtained from the MARG. This figure was created in Matlab after the contents of the CSV file were read into the workspace. Here, we display the evolution through time of the three accelerometer channel values (acc_x, acc_y, and acc_z), the three gyroscope channel values (gyro_x, gyro_y, and gyro_z), and the three magnetometer channel values (mag_x, mag_y, and mag_z) in the top three subplots, respectively. Finally, the bottom subplot displays the evolution of the four components of the quaternion orientation calculated by the Trio system (cam_qx, cam_qy, cam_qz, and cam_qw). It is in this plot that the timing of the poses can best be recognized. Poses were identified by their numbers (underscored), using blue font for Poses 1–5 held at location (A) and red font for Poses 6–10 held at location (B), where the magnetic field was distorted. It can be seen that while the accelerometer and gyroscope signals during the 2nd part of the record are very similar to those in the 1st part (since the sequence of rotations executed in (B) was the same as those executed in (A)), the magnetometer signals for Poses 6–10 are clearly distorted with respect to those observed for Poses 1–5, as expected.

Figure 4 shows all the information generated by the Trio system. The top subplots display the evolution through time of the position coordinates (pos_x, pos_y, and pos_z). The second subplot displays (again) the time evolution of the components of the quaternion calculated by the Trio system (cam_qx, cam_qy, cam_qz, and cam_qw), with the same labeling of poses used in Figure 3. The next subplot displays the values of the “isTracked” flag. This flag recorded a value of 1 (all reflective markers were observable by the Trio system) most of the time, with only a few occurrences of the value 0, which identifies the few sampling instants in which not all three of the reflective markers were observable. These occurrences were rare during the recordings. For example, in the file visualized in Figure 3 and Figure 4, isTracked was 1 for 98.65% of the samples. This figure also includes, at the bottom, the time evolution of the four components of the orientation quaternion calculated by the onboard Kalman filter implemented in the Yost Labs 3–Space MARG.

Figure 5 shows an example of the kind of assessment of MARG orientation estimators that can be performed using the files in FIUMARGDB. At the top, the quaternion components from the Trio system are shown. These orientations, expressed as quaternions, can be taken as the orientation “ground truth”. The middle subplot in the figure shows the evolution of the quaternion components generated by the onboard Kalman filter. It can be observed that during the first part of the recording both estimates are very similar. However, when the MARG was translated to location (B), where the magnetic field was distorted (Poses 6–10), the altered magnetometer readings that can be seen in Figure 3 negatively impact the performance of the Kalman filter orientation estimation. As a result, the Kalman filter components take on erroneous values, whereas the estimation process in the Trio system is not affected and produces a very similar sequence of orientation estimates for Poses 6–10 as that produced for Poses 1–5 in location (A).

From the series of four-valued quaternions from Trio and the series of four-valued quaternions from the Kalman filter, is possible to derive a series of “quaternion distance” measurements throughout the complete record. This can be obtained through the “dist” command (angular distance in radians), which, according to Matlab, “returns the angular distance in radians between two quaternions” [33]. This instantaneous error measure is similar to the one used in [24].

The bottom subplot in Figure 5 displays the evolution through time of the “quaternion distance” (already converted to degrees) between the orientation estimates from the Trio system and the onboard Kalman filter. It is easy to recognize that the distance increases significantly during the interval of the recording in which the MARG was at location (B). Therefore, if the orientation estimate from the Trio system is considered the “ground truth”, this last graph can be interpreted as indicating that the performance of the Kalman filter in MARG orientation estimation degraded significantly while the MARG was in the magnetically distorted environment.

**Figure 6:**
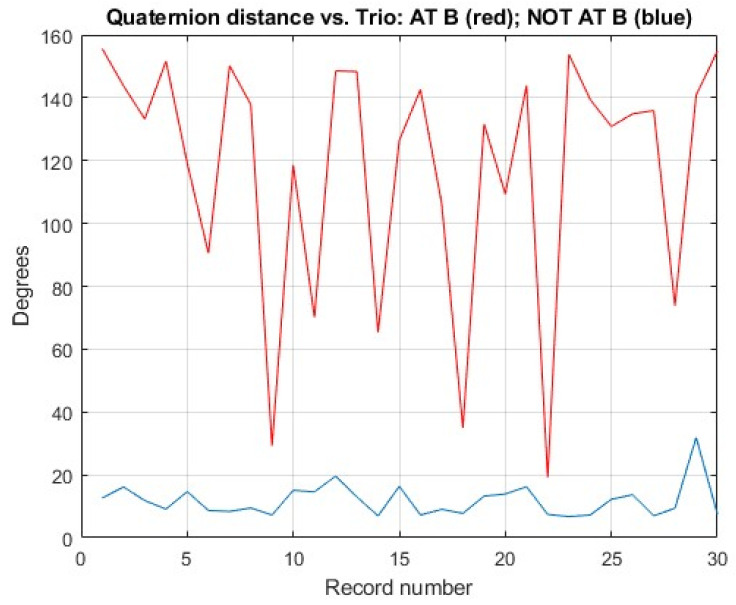
RMS values of quaternion distance (Trio vs. onboard Kalman filter) for the recordings in the FIUMARGDB. The red trace represents the RMS value calculated only in the interval when the MARG was in the neighborhood of Location B (at B). The blue trace represents the RMS value of the remainder of the record (not at B).

Figure 6 shows the root mean square (RMS) value of the quaternion distance in degrees (Trio vs. Kalman filter) for each of the recordings in the FIUMARGDB dataset. The red trace is the RMS value incurred only while the MARG was in the neighborhood of location (B), as identified by negative values in the position coordinate TX (“at B”). The blue trace is for the RMS value computed in the remainder of the recording run (“not at B”). While the RMS of the quaternion distance “at B” varies from record to record, we can see that it is typically much higher than the RMS “not at B”. The average and standard deviation values are 118.0371° and 39.4526°, respectively, for at B and 11.8065° and 5.2525°, respectively, for not at B, which suggests that most of the quaternion distance resulted from the lack of resilience of the orientation estimation algorithm under magnetically distorted conditions.

It should be pointed out that for our recordings we configured the AHRS filter onboard the Yost 3-Space module to execute the simple implementation of a Kalman filter, just as a basic item for comparison. The Yost 3-Space module is also capable of implementing faster orientation filters, such as “Q-COMP (quaternion complementary) filter” and “Q-GRAD (quaternion gradient descent) filter”, instead of the Kalman filter algorithm [29].

In the repository, within the folder “Extra_Files_2”, we provide three recordings from runs in which the MARG was configured to implement the Q-COMP filter, and three recordings from runs in which the MARG was configured to implement the Q-GRAD filter. For each of those groups of three records, and for the first three records from our main dataset (rec01.csv, rec02.csv, and rec03.csv), we computed the root mean square (RMS) value of the quaternion distance (e.g., bottom plot in Figure 5 for the Kalman filter orientations from rec03.csv) over the complete run. Table 5 shows the means and standard deviations we found for each type of filter (the values for Kalman filter, KF, refer to RMS computed from files rec01.csv, rec02.csv, and rec03.csv).

We can see in Table 5 that Q-GRAD recorded a slightly lower RMS value of the quaternion distance and Q-COMP recorded a slightly higher RMS value of quaternion distance with respect to the Kalman filter used for all the recordings (30) in our main dataset. Appendix B shows representative plots of quaternion components and quaternion distance (with respect to the Trio orientations) for Q-GRAD and Q-COMP, which can be compared to the lower two plots in Figure 5. In the Q-GRAD plots, we can see that the orientation estimates deteriorate more gradually than for Q-COMP. This might be the reason for the lower RMS value of the quaternion distance displayed by Q-GRAD.

## 4. Discussion

The main objective of our development of FIUMARGDB was to create a series of MARG data files (i.e., readings from the MARGs tri-axial accelerometer, tri-axial gyroscope, and tri-axial magnetometer) that would be accompanied by the corresponding series of orientation measurements (obtained by the Trio optical motion capture system) which could be considered “ground truth” values of MARG orientation. In particular, we sought to create such combined MARG–ground truth orientation recordings in the following context:

- The MARG signals should come from a low-cost, commercially available MEMS MARG module, as it is for these modules that the signal processing requirements are most challenging but the potential rewards are most promising.
- The environmental context and movements carried out by the MARG module should be similar to those a MARG module may experience in its application in body movement (e.g., hand and fingers movement) tracking for the purpose of human–computer interaction (as that is our area of work [12,34,35]).

Accordingly, we used the 3-Space Attitude and Heading Reference System (AHRS)/inertial measurement unit (IMU) from Yost Labs (https://yostlabs.com/, accessed on 15 February 2023). Yost Labs offers the same basic MARG system in a variety of packages and with different forms of communication to a host. The basic versions can be affordable (particularly if purchased in medium or large quantities). However, we employed the 3-Space MARG version that communicates wirelessly to a PC host to avoid the disruptive tethering effect that a wired connection from the sensor could have.

### 4.1. Discussion of the Main Set of Recordings

To explore the types of circumstances in which the MARG may operate as part of a human–computer interaction system, we asked a number of volunteer subjects to perform the same pre-defined sequence of translations and rotations with the MARG at two locations. The magnetic field at the first location, (A), was assumed to be the undisturbed local geomagnetic field. In contrast, magnetic disrupters (described in Section 2.2.3) were placed under the second location, (B), so that the magnetic field sensed by the MARG would be distorted by design. We aimed at capturing the different movement characteristics that could be expected from diverse human operators (e.g., speeds, specific trajectories, continuity of motion, etc.) by recording the same sequence of actions executed by several volunteers.

The generation of independently obtained orientation estimates was achieved by affixing the 3-Space MARG module to a plastic “rigid body hand (emulator)” with three reflective markers, as recommended by the manufacturers of the V120:Trio tracking system, in order to generate orientation measurements of the rigid body defined by the markers.

The appropriateness of using the orientations calculated from the Trio system as “ground truth” to assess the effectiveness of MARG orientation estimation algorithms stems from the documented position tracking accuracy of this type of optical motion capture (OMC) system, and from the fact that the orientation computation procedures from marker positions are not iterative (as opposed to most MARG orientation estimation approaches). The manufacturer of the VT120:Trio system makes the general statement (https://optitrack.com/applications/movement-sciences/#accuracy, accessed on 15 February 2023) that “OptiTrack systems typically produce less than 0.2 mm of measurement error, even across large tracking areas—even of those 10,000 sq ft or more. In smaller measurement areas, OptiTrack systems regularly produce errors of 0.1 mm or less”, which references the 2017 study by Aurand et al. [36], where they assessed the position tracking accuracy of an OptiTrack system that employed 42 cameras for tracking a large volume of 135 m^3^. Aurand et al. concluded that “the OMC system demonstrated submillimeter mean accuracy at every location in the capture volume, and error was found to be less than 200 μm in 97% of the capture volume (using all 42 cameras)”, also indicating that the errors were found to be less than 200 μm in 91% of the capture volume if only 21 cameras were involved. While their study dealt with a much larger capture volume that necessitates the involvement of larger numbers of cameras, they also commented that the errors measured in a study by Eichelberger et al. [37], which “measured inter-marker distance using 6–10 Vicon cameras within a 13.2 m^3^ (5.5 m × 1.2 m × 2 m) capture volume…were of the same order of magnitude as the current study”. This level of position tracking accuracy in the same type of optical motion capture systems as the V120:Trio supports the use of its orientation estimates as “ground truth”.

While the orthogonal Cartesian axis frames used by the Trio system and by the 3-Space MARG are not the same (the former is right-handed and the latter is left-handed), care was exercised to set the initial orientation of the MARG so that each of its axes would be precisely parallel to one of the Trio axes (e.g., to make x parallel to TX and y parallel to TY in Figure 1). If, nonetheless, for a particular recording, the initial direction of the MARG x axis was inadvertently misaligned with respect to TX by a small rotation, this “rotational offset” may be compensated for by applying a compensatory rotation (encoded as a quaternion, ***q***_C_) to the results of a MARG-based orientation estimate, such as, for example, the Kalman filter estimate, ***q***_Kalman_:

(5) $$q_{KalmanComp}=q_{C}⊗q_{Kalman}$$

where ***q***_Kalman_ is the Kalman filter quaternion at any given sampling instant (whose components in the file are ss_qx, ss_qy, ss_qz, and ss_qw according to Table 4), ***q***_C_ is the (constant) “compensatory quaternion”, ⊗ indicates the quaternion product, and ***q***_KalmanComp_ is the compensated quaternion for that sampling instant. Appendix A describes how the values of the components of ***q***_C_ can be obtained from an FIUMARGDB file and the matrix equation needed for the compensation of each MARG-based orientation quaternion.

### 4.2. Supplementary Recordings: Reverse Location Itinerary and Alternative Disrupter Placements

Beyond the main set of recordings that constitute our dataset, we have sought to provide users with two critical variations of the manipulations of the MARG and the location of the magnetic disrupter.

#### 4.2.1. Reverse Itinerary Recordings

We have included (in the folder Extra_Files_3) seven recordings in which the setup is the same as in the main recordings of the dataset, except that the locations are visited in a reverse circuit. That is, at the beginning of the recording, the MARG is picked up from (H) and it is translated, without rotation, to location (B), where there is a disrupted magnetic environment, *first*. At (B), the same sequence of poses as usual (Poses 6, 7, (6), 8, (6), 9, (6), 10, (6)) are held *and then* the MARG is translated, without rotation, to location (A), where the usual poses (Poses 1, 2, (1), 3, (1), 4, (1), 5, (1)) are held. Finally, the MARG is returned to the home location (H). These recordings may be helpful in assessing the capability of a given orientation estimator to “recover” and provide correct orientation estimates in (A) if the estimates generated first in (B) were erroneous. These recordings, with names that start with “rer” (last r meaning “reverse”) instead of “rec”, can also be visualized with the same Matlab functions and scripts as provided for the standard recordings (e.g., “rec03.csv”).

#### 4.2.2. Alternative Positioning of the Magnetic Disruptor

**Figure 7:**
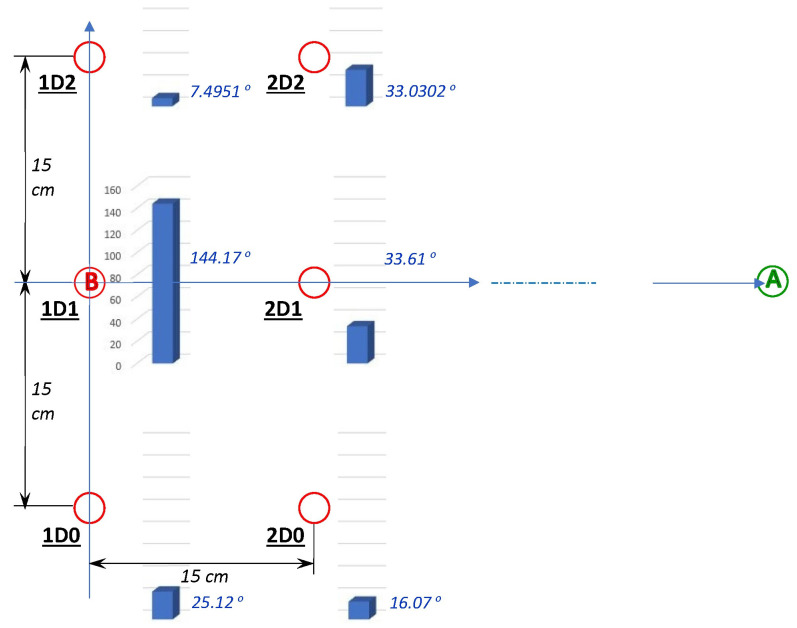
Alternative locations of the magnetic disrupter and corresponding effects in Location (B). (The circled A and B letters represent Locations (A) and (B), respectively.) The red circles are the locations of the magnetic disrupter labeled as XDY, where 1D1 is the standard placement at (B). The heights of the blue prisms represent the RMS value of Kalman filter estimation quaternion distance with respect to the Trio orientation estimation (in degrees) while the MARG was hovering over location (B) and Poses 6–10 were held according to the standard sequence.

We also provide five sets of three recordings each in which the magnetic disruptor cluster shown in Figure 1 was not placed directly under location (B), but instead it was placed under locations 15 cm to the east of (B) and/or shifted by −15 cm, 0 cm, or +15 cm from south to north with respect to the original location under (B). Figure 7 shows the alternative locations of the magnetic disrupter xDy (where x = 1 or 2 and y = 0, 1, or 2). The 15 recordings are included in the folder Extra_Files_4 of the dataset, and their names follow the convention xDy-n.csv, where n is 1, 2, or 3. For completeness, this folder also contains three files from the main group of files, which can be used for comparison purposes: rec01.csv = 1D1-1.csv, rec02.csv = 1D1-2.csv, and rec03.csv = 1D1-3.csv. For a first assessment of the impact of magnetic disrupter placement on MARG orientation estimate at Location (B), we evaluated the RMS value of the quaternion difference (Kalman filter vs. Trio estimates) just while the MARG was at location (B) for each of the 18 files. The itinerary for all these files was the “standard itinerary”, i.e., the one followed during the recording of all the files in the main dataset: Location (H)–Location (A)–Location (B)–Location (H). (We identified that the interval in which the MARG was in the neighborhood of location (B) with the interval of the recording in which the coordinate TX of the position reported by Trio was negative.) Figure 7 shows vertical bars with the average RMS quaternion difference (Trio vs. Kalman filter) observed at location (B) when the magnetic disrupter was under each of the six locations.

The additional 15 recordings may be used by orientation estimation algorithm designers to expose their algorithms to magnetic distortions that have different degrees of strength and are centered at locations around the point of MARG test. The heights of the blue prisms in Figure 7 followed a configuration that was partially expected. Except for the placement of the disrupter right below the area where the MARG operated (placement 1D1), the impact on the performance of the onboard Kalman filter was greatest when the disrupter was placed 15 cm to the east (2D1) or to the south (1D0) of the testing point where the MARG was operated (Location B). There was a smaller impact when the disrupter was placed 21.21 cm in a southeast direction from B (2D0). However, the impacts on the performance at (B) when the disrupter was placed at 1D2 and 2D2 did not match the results for placement in 1D0 and 2D0. This may be, however, a result of the asymmetrical configuration of the disrupter, which has a “thick” steel bar across its south boundary, without a corresponding bar across its north boundary. In any case, Figure 7 confirms that the additional 15 recordings in folder Extra_Files_4 will offer a wider variety of magnetic disruptions that algorithm designers can use for testing. Ultimately, a truly robust orientation estimation algorithm should not degrade under any of the conditions represented in the 15 additional files and it should also be resilient to avoid degradation under the stronger magnetic disruptions involved in the standard files (rec01.csv through rec30.csv) of our dataset.

## 5. Conclusions

This paper has presented the FIUMARGDB dataset of MARG signals accompanied by “ground truth” orientation estimation quaternions. This dataset is meant to facilitate the benchmarking of orientation estimation algorithms that use signals from the accelerometers, gyroscopes, and magnetometers in a MARG module to compute the orientation of the module, typically as a quaternion. Benchmarking has becoming increasingly important because it has been found that different orientation estimation algorithms may be more severely affected in their performance under certain circumstances. FIUMARGDB was created specifically to expose orientation estimation algorithms to operating environments with and without magnetic distortion.

FIUMARGDB was developed for the benchmarking of orientation algorithms in a context that might be similar to the one experienced by MARG modules used in human–computer interaction devices. Accordingly, all the records were obtained while the MARG module was moved by a human subject with their dominant hand. This defines the range and speed of MARG rotations and translations that were recorded. Similarly, we included recordings created with the participation of multiple human volunteers to capture the corresponding possible variations in speed, trajectory, continuity of movement, and steadiness of poses held.

We selected a basic, low-cost MARG module for the recordings, since the required processing of signals from this type of MARG might be the most challenging. At the same time, very small and low-cost MARG modules will be the best suited type for developing some human–computer interaction devices, such as an instrumented glove to track the orientation and configuration of the hand of a computer user (which may require the inclusion of many MARG modules in the glove). Similar priorities may apply to MARG usage in the fields of human motion studies and motor rehabilitation.

The FIUMARGDB dataset and Matlab programs for its use can be accessed through this URL: https://github.com/LABDSP/FIUMARGDB_marg_signals_and_reference_orientations.git (accessed on 15 February 2023).

## Figures and Tables

**Table 1:** Direction of inertial frame axes (for orientations) with respect to labels (H), (A), and (B).

AXIS	AXIS DIRECTION
x AXIS	Parallel to the (B) to (A) direction, positive towards (A)
y AXIS	Parallel to the floor-to-ceiling direction, positive towards the ceiling
z AXIS	Parallel to the (A) to (H) direction, positive towards (H)

**Table 2:** Direction of V120:TRIO axes (for positions) with respect to labels (H), (A), and (B).

AXIS	AXIS DIRECTION
TX AXIS	Parallel to the (B) to (A) direction, positive towards (A)
TY AXIS	Parallel to the floor-to-ceiling direction, positive towards the ceiling
TZ AXIS	Parallel to the (H) to (A) direction, positive towards (A)

**Table 3:** Sequence of steps in each recording run.

Sequence Step	Location	Rotation	Resulting Pose
1	H	(Initial location and pose for the task)	1 <Default Pose>
2	(to) A	After translation H to A, yields	1
3	A	+90° Z Axis, yields	2
4	A	−90° Z Axis, yields	1
5	A	+90° X Axis, yields	3
6	A	−90° X Axis, yields	1
7	A	+90° Y Axis, yields	4
8	A	−90° Y Axis, yields	1
9	A	−45° Y Axis and + 90° X Axis, yields	5
10	A	+45° Y Axis and − 90° X Axis, yields	1
11	(to) B	Just translation A to B	6 (same orientation as 1)
12	B	+90° Z Axis, yields	7
13	B	−90° Z Axis, yields	6
14	B	+90° X Axis, yields	8
15	B	−90° X Axis, yields	6
16	B	+90° Y Axis, yields	9
17	B	−90° Y Axis, yields	6
18	B	−45° Y Axis and + 90° X Axis, yields	10
19	B	+ 45° Y Axis and − 90° X Axis, yields	6
20	(to) H	Just translation back to H	1

**Table 4:** Organization of the files in FIUMARGDB.

Entity (Units)	Column	Data (Header)
Timestamp (ms)	1	Timestamp
Trio position (m)	2	pos_x
	3	pos_y
	4	pos_z
Trio orientation (normalized unit quaternion)	5	cam_qx
	6	cam_qy
	7	cam_qz
	8	cam_qw
Kalman filter orientation (normalized unit quaternion)	9	ss_qx
	10	ss_qy
	11	ss_qz
	12	ss_qw
Gyroscope readings (rad/s)	13	gyro_x
	14	gyro_y
	15	gyro_z
Accelerometer readings (g)	16	acc_x
	17	acc_y
	18	acc_z
Magnetometer readings (Gauss)	19	mag_x
	20	mag_y
	21	mag_z
Confidence Factor	22	stillness
isTracked	23	isTracked

**Table 5:** RMS values of quaternion distance using various onboard orientation filters.

	Q-GRAD	Kalman Filter	Q-COMP
Q distance mean (°)	83.8225	96.8412	100.6647
Q distance std. dev. (°)	8.5298	6.5633	2.1149

**Figure A1:**
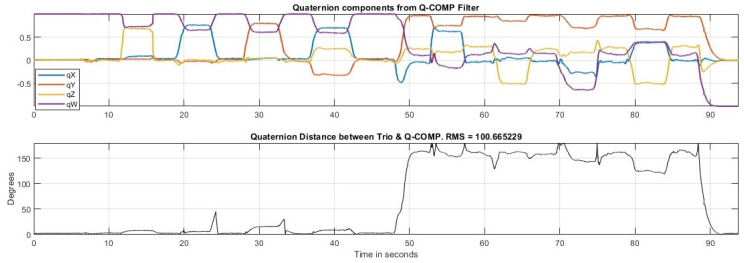
(**Top**) evolution of the quaternion components generated by the Q-COMP onboard filter. (**Bottom**) evolution of the quaternion distance between the Trio quaternion result and the Q-COMP quaternion result.

**Figure A2:**
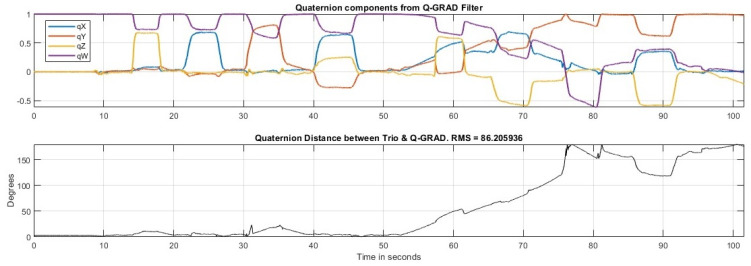
(**Top**) evolution of the quaternion components generated by the Q-GRAD onboard filter. (**Bottom**) evolution of the quaternion distance between the Trio quaternion result and the Q-GRAD quaternion result.

## Data Availability

The record files of the FIUMARGB dataset described in the paper, as well as Matlab programs for their use, can be accessed through this URL: https://github.com/LABDSP/FIUMARGDB_marg_signals_and_reference_orientations.git (accessed on 15 February 2023).

## Supplementary Materials

The following supporting information can be downloaded at: https://www.mdpi.com/article/10.3390/s23083786/s1, Video: FIUMARGDB_sequence.mp4.

## Appendix A

In reference to Figure 1, where the blue coordinate frame (x, y, z) corresponds to the initial orientation (at startup) of the MARG and the red coordinate frame (TX, TY, TZ) corresponds to the Trio system, efforts were made in the recording of the files to set the initial orientation of the MARG so that x and TX would be parallel, and y and TY would be parallel too. Most MARG orientation algorithms will adopt the blue frame as the “inertial frame”, reporting future MARG orientations with respect to it. Under those circumstances, the conversion equations shown in Section 2.2.2 will adjust the quaternion results of Trio orientation estimation to be directly comparable to the quaternion MARG orientation estimates.

If, on the other hand, a misalignment of the red and blue frames exists at startup, such that, for example, the x axis is rotated with respect to the TX axis, the MARG orientation estimates will likely be referenced to that inertial frame, which has a constant “rotation offset” with respect to the estimates generated by the Trio system.

Vince [38] showed that a quaternion, ***q***_3_, that represents the compounding of the rotations represented by ***q***_1_ first, followed by ***q***_2_ afterwards, is obtained simply through this quaternion product:

(A1) $$q_{3}=q_{2}⊗q_{1}$$

Therefore, the “rotation offset” can be removed by obtaining a new, compensated orientation estimate, e.g., ***q***_KalmanComp_, which is the composition of the original MARG-based estimate, e.g., ***q***_Kalman_, modified by an appropriate compensation quaternion, ***q***_C_:

(A2) $$q_{KalmanComp}=q_{C}⊗q_{Kalman}$$

To figure out the values needed in the components of ***q***_C_, we focus on the fact that Equation (A2) must be true throughout the complete record, but, in particular, at startup, when the MARG-based algorithms would indicate a MARG orientation encoded by the quaternion ***q***_Kalman_ = [ss_qx, ss_qy, ss_qz, ss_qw] = [0, 0, 0, 1], which indicates “no rotation”, i.e., there is no rotation (yet) mediating between the MARG body frame and the inertial frame. At startup, the Trio orientation in general will be ***q***_Trio0_ = [cam_qx0, cam_qy0,cam_qz0,cam_qw0].

(Note: Since the equations in Appendix A are based on those by Vince, the scalar part of all quaternions in this appendix are placed first, as opposed to the convention used in the body of the paper, where the scalar is shown as the last (fourth) component of the quaternions.) Vince [38] indicated that for two quaternions

(A3) $$q_{a}=\left[s_{a},a\right]=\left[s_{a},x_{a}i+y_{a}j+z_{a}k\right]$$

(A4) $$q_{b}=\left[s_{b},b\right]=\left[s_{b},x_{b}i+y_{b}j+z_{b}k\right]$$

the product can be computed through this matrix product (note *s*_a_ and *s*_b_ are the scalar components of quaternions ***q***_a_ and ***q***_b_, respectively):

(A5) $$\begin{array}{c} q_{a}⊗q_{b}=\left[s_{a},a\right]⊗\left[s_{b},b\right]\\ =[s_{a}s_{b}-a\cdot b,s_{a}b+s_{b}a+a⨯b]\\ \begin{array}{cc} =\left[\begin{array}{cc} \begin{array}{cc} s_{a} & -x_{a}\\ x_{a} & s_{a} \end{array} & \begin{array}{cc} -y_{a} & -z_{a}\\ -z_{a} & y_{a} \end{array}\\ \begin{array}{cc} y_{a} & z_{a}\\ z_{a} & -y_{a} \end{array} & \begin{array}{cc} s_{a} & -x_{a}\\ x_{a} & s_{a} \end{array} \end{array}\right] & \left[\begin{array}{c} \begin{array}{c} s_{b}\\ x_{b} \end{array}\\ \begin{array}{c} y_{b}\\ z_{b} \end{array} \end{array}\right] \end{array} \end{array}$$

Therefore, substituting ***q***_C_ = [qC_x, qC_y, qC_z, qC_w], the compensating quaternion we want to find for ***q***_a_, and the initial MARG-based orientation quaternion, [0, 0, 0, 1], for ***q***_b_, their product should yield the initial ***q***_Trio0_ quaternion:

(A6) $$\left[\begin{array}{cc} \begin{array}{cc} qC\_w & -qC\_x\\ qC\_x & qC\_w \end{array} & \begin{array}{cc} -qC\_y & -qC\_z\\ -qC\_z & qC\_y \end{array}\\ \begin{array}{cc} qC\_y & qC\_z\\ qC\_z & -qC\_y \end{array} & \begin{array}{cc} qC\_w & -qC\_x\\ qC\_x & qC\_w \end{array} \end{array}\right]\left[\begin{array}{c} \begin{array}{c} 1\\ 0 \end{array}\\ \begin{array}{c} 0\\ 0 \end{array} \end{array}\right]=\left[\begin{array}{c} \begin{array}{c} cam\_qw0\\ cam\_qx0 \end{array}\\ \begin{array}{c} cam\_qy0\\ cam\_qz0 \end{array} \end{array}\right]$$

That is:

qC_w = cam_qw0; qC_x = cam_qx0; qC_y = cam_qy0; qC_z = cam_qz0.

In other words, the components of the “compensating quaternion”, ***q***_C_, are just the components of the first quaternion generated by the Trio system at startup. Once those values are read from the first row of any given file in FIUMARGDB, the compensation of each MARG estimated quaternion (for example the values ss_qx, ss_qy, ss_qz, and ss_qw from the Kalman filter) only need to be pre-multiplied by the corresponding 4-by-4 matrix to yield the four values of the compensated quaternion:

(A7) $$\begin{array}{cc} \left[\begin{array}{cc} \begin{array}{cc} qC\_w & -qC\_x\\ qC\_x & qC\_w \end{array} & \begin{array}{cc} -qC\_y & -qC\_z\\ -qC\_z & qC\_y \end{array}\\ \begin{array}{cc} qC\_y & qC\_z\\ qC\_z & -qC\_y \end{array} & \begin{array}{cc} qC\_w & -qC\_x\\ qC\_x & qC\_w \end{array} \end{array}\right] & \left[\begin{array}{c} \begin{array}{c} ss\_qw\\ ss\_qx \end{array}\\ \begin{array}{c} ss\_qy\\ ss\_qz \end{array} \end{array}\right] \end{array}=\left[\begin{array}{c} \begin{array}{c} ss\_qwComp\\ ss\_qxComp \end{array}\\ \begin{array}{c} ss\_qxComp\\ ss\_qwComp \end{array} \end{array}\right]$$

where the elements in the last column vector to the right in the above equation can be used to substitute the original elements of the quaternion encoding the original (uncompensated) result of the MARG-based orientation estimation.

It can be noticed that, for the file visualized in Figure 4, the initial Trio quaternion has components cam_qx0 = 0.0032, cam_qy0 = −0.0280, cam_qz0 = −0.0222, and cam_qw0 = 0.9993. Therefore, the compensating matrix in Equation (A7) will be very similar to a 4-by-4 identity matrix, which means that the differences between the compensated quaternion and the original quaternion will probably be negligible.

## Appendix B

We recorded six additional files with the standard setup and performing the same standard sequence of steps indicated in Table 3 but choosing different onboard orientation filters. A summary of the performance of those filters, in comparison with the performance of the Kalman filter chosen for the basic set of records in our dataset, is provided in Table 5.

Here, we present plots of the evolution of the quaternion components and the evolution of the quaternion distance (with respect to the Trio orientation estimation, acting as a ground truth) for the two alternative orientation filters. These plots also display the numerical value of RMS of the quaternion distance for the complete record (in degrees).

Figure A1 shows the results for a representative file in which the Q–COMP filter was chosen (file qcomp2.csv). In this case, the RMS value was 100.66°. The traces in this figure can be compared with the lower two subplots of Figure 5, which were obtained from file rec03.csv, in which the Kalman filter was selected. The RMS value for that case was 90.03°.

Figure A2 shows the results for a representative file in which the Q–GRAD filter was chosen (file qgrad1.csv). In this case, the RMS value was 86.20°. This slightly lower level of error may result from the more gradual deterioration of the orientation estimate that seems to take place when the Q–GRAD filter was used. However, these observations seem to confirm that the orientation estimates from the three types of filters degrade noticeably when the MARG is operating in a magnetically distorted area.
